# Effects of Three Different Fibrates on Intrahepatic Cholestasis Experimentally Induced in Rats

**DOI:** 10.1155/2013/781348

**Published:** 2013-08-12

**Authors:** Alaa El-Sisi, Sahar Hegazy, Eman El-Khateeb

**Affiliations:** ^1^Pharmacology and Toxicology Department, Faculty of Pharmacy, Tanta University, Tanta 31111, Egypt; ^2^Clinical Pharmacy Department, Faculty of Pharmacy, Tanta University, Tanta 8310, Egypt

## Abstract

*Background*. Activation of PPAR**α** modulates cholesterol metabolism and suppresses bile acid synthesis. This study aims to evaluate the effect of PPAR**α** agonists, fenofibrate, bezafibrate, and gemfibrozil, on acute cholestasis induced by ethinylestradiol (EE) plus chlorpromazine (CPZ) in rats. *Method*. 100 male albino rats (150–200 gm) were divided randomly into 10 equal groups. Control group received 1% methylcellulose vehicle; disease group received CPZ plus EE for 5 consecutive days; four groups received either ursodeoxycholic acid, fenofibrate, bezafibrate, or gemfibrozil for 7 days; 2 days before EE + CPZ, three other groups received one of the three fibrates after GW6471, a selective PPAR**α** antagonist in addition to EE + CPZ. The final group received GW6471 alone. *Results*. The three fibrates showed marked reduction (*P* < 0.05) in serum levels of ALP, GGT, ALT, AST, total bile acids, bilirubin, TNF**α**, and IL-1**β** and in hepatic malondialdehyde level as well as a significant increase in bile flow rate (*P* < 0.05) in addition to improvements in histopathological parameters compared to diseased group. In groups which received GW6471, these effects were completely abolished with fenofibrate and partially blocked with bezafibrate and gemfibrozil. *Conclusion*. Short-term administration of fibrates to EE/CPZ-induced intrahepatic cholestatic rats exerted beneficial effects on hepatocellular damage and apoptosis. Fenofibrate anticholestatic effect was solely PPAR**α** dependent while other mechanisms played part in bezafibrate and gemfibrozil actions.

## 1. Background

Cholestasis is defined as a disturbance of bile secretion that can result from a functional defect in bile formation at the level of hepatocytes or from impaired bile secretion and flow at the bile duct level [1]. It results in intrahepatic accumulation of cytotoxic bile acids, which cause liver damage ultimately leading to biliary fibrosis and cirrhosis and ultimately end-stage liver disease requiring liver transplantation. Cholestatic liver injury is counteracted by a variety of adaptive hepatoprotective mechanisms including alterations in bile acid transport, synthesis, and detoxification [2]. Because the intrinsic adaptive response to bile acids cannot fully prevent liver injury in cholestasis, therapeutic targeting of many nuclear receptors *via* specific and potent agonists may further enhance the hepatic defense against toxic bile acids. Therefore nuclear receptors (NRs) are promising therapeutic targets for cholestatic liver diseases [3].

Peroxisome proliferator-activated receptor alpha (PPAR*α*), farnesoid X receptor (FXR), pregnane X receptor (PXR), and hepatic nuclear factor 4*α* (HNF4*α*) are examples of NRs playing vital role in bile acid homeostasis with interplay among these receptors in this process. Therefore ligands of these receptors are thought to be potential treatments of cholestatic liver diseases [3]. In addition, there is crosstalk between the PPAR*α* and FXR transcriptional pathways because PPAR*α* is an FXR target gene harboring an FXR response element in its gene promoter [4].

Several animal models of intrahepatic cholestasis which simulate human cholestatic diseases are adopted such as oral contraceptive-induced cholestasis using ethinylestradiol [5].

Estrogens are well known to cause reversible intrahepatic cholestasis in humans and rodents. Intrahepatic cholestasis occurs in susceptible women during pregnancy or due to administration of oral contraceptives and postmenopausal hormone replacement therapy [6]. In rats, the administration of ethinylestradiol, a synthetic estrogen, causes a reduction in bile flow and an impairment of several transport mechanisms in both basolateral and canalicular hepatocyte membranes [5].

Chlorpromazine (CPZ) is a tricyclic antidepressant that has been used as a sedative and antiemetic and for the management of psychotic disorders. CPZ and its hydroxylated metabolites cause irreversible inhibition of bile flow as they decrease Na^+^/K^+^-ATPase and Mg_2_^+^-ATPase cation pumping in a dose-dependent fashion causing cholestatic hepatitis [7]. 

Fibrates like fenofibrate, bezafibrate, or gemfibrozil are already commercially available drugs in the treatment of hyperlipidemia and are generally effective in lowering elevated plasma triglyceride and cholesterol levels [8]. They exert multiple effects on lipid metabolism pathways by activating peroxisome proliferator-activated receptor alpha (PPAR*α*), one of nuclear receptors which control gene expression through peroxisome proliferators response elements (PPREs) [9]. 

Indeed, fibrates suppress bile acid synthesis, the major pathway of cholesterol elimination from the body [10], and regulate detoxification and biliary phospholipid secretion by induction of their output through multidrug resistance transporter-2 (Mdr2) activation [11]. Induction of PPAR*α* increases the size and the number of hepatocytes within the first few days of exposure. During this short exposure time, spontaneous hepatocyte apoptosis is suppressed within the intact liver [12]. 

Due to these effects, the present study was conducted to investigate the effect of three different fibrates in experimentally induced intrahepatic cholestasis and to determine the role of PPAR*α* receptor agonism in this effect if present. 

## 2. Materials and Method

### 2.1. Chemicals

Lopid; 300 mg gemfibrozil (Pfizer Co., Egypt). Fenofibrate (Sigma Pharm. Co., Egypt). Bezafibrate (Epico Pharm. Co., Egypt). Ethinylestradiol (Sigma-Aldrich Corp., St. Louis, MO, USA). Neurazine; 100 mg chlorpromazine (Misr Co., Egypt). GW6471 (Tocris bioscience, USA).

All other chemicals used were of analytical grade.

### 2.2. Animals and Treatment

100 male albino rats (150–200 gm) were randomized into ten groups of ten rats each. Rats were obtained from the animal house of the National Research Center (NRC), Egypt. They were housed under controlled environmental conditions and had free access to standard chow and water.

Group 1 (control group) was given a vehicle (1% methyl cellulose) by oral gavage for 7 consecutive days. Group 2 was given 17*α*-ethinylestradiol EE (5 mg/kg/d) S.C. + oral chlorpromazine CPZ (30 mg/kg/d) for 5 consecutive days. Groups 3 to 6: animals were cotreated EE & CPZ with either fenofibrate (200 mg/Kg/day), bezafibrate (200 mg/Kg/day), gemfibrozil (120 mg/Kg/day), or UDCA (100 mg/Kg/day) suspended in 1% methylcellulose or in saline for UDCA and were administered by oral gavages for 7 consecutive days (2 days before EE & CPZ administration). Groups 7 to 9 were cotreated EE & CPZ with GW6471 (1 mg/kg/day) i.p as antagonist of PPAR alpha receptors, 30 min before fenofibrate, bezafibrate, or gemfibrozil. The last group was treated with GW6471 (1 mg/Kg/day) i.p for 7 consecutive days.

At the end of the treatment period, blood samples were withdrawn by heart puncture under ether anesthesia to assess biochemical parameters. Thereafter, the animals were killed by cervical dislocation. The livers were dissected out, cut into two parts: the first was kept deep frozen at −20°C for assessment of malondialdehyde level (MDA). The other part was preserved in 10% neutral formalin and used for the histopathological and immunohistochemical examinations.

### 2.3. Biochemical Analysis

- *Measurement of liver enzyme activities:*
  - the serum enzyme activities of ALT & AST were measured colorimetrically according to the method of Reitman and Frankel [13], using Biodiagnostic kits, Egypt.
  - The serum enzyme activities of ALP & GGT were measured colorimetrically according to the kinetic method of IFCC (International Federal of Clinical Chemistry) recommendations for ALP, using Greiner diagnostic kits, Germany.
- *Measurement of serum total and direct bilirubin* colorimetrically according to the method of Walters and Gerarde [14], using Biodiagnostic kits, Egypt.
- *Measurement of serum total bile acids (TBA)* colorimetrically using Diazyme laboratories kits, Poway, CA, USA.
- *Measurement of hepatic malondialdehyde (MDA)* level colorimetrically according to Yoshioka et al., chemical method [15].

The optical density for all these parameters was read at 405 nm using Shimadzu UV-PC 1601, Japan spectrophotometer.

(v) * Measurement of serum cytokines levels:*
  - the serum level of TNF*α* was measured colorimetrically using Assaypro ELISA kit, USA.
  - The serum level of IL-1*β* was measured colorimetrically using Cusabio Biotech ELISA kit, China.

The optical density was read at 450 nm using microplate reader (LMR-9602, U.S.A).

### 2.4. Bile Flow Rate Measurement

Bile collection started between 9:00 and 11:00 a.m. to minimize influence of circadian variations. Animals were anesthetized with a single dose of urethane (1 g/kg rat b.wt intramuscularly.) A middle abdominal incision was made, and the common bile duct was cannulated using a PE-10 polyethylene tubing. Body temperature was maintained at 37.0–38.5°C with a warming lamp to prevent hypothermic alterations of bile flow. Bile flow rate was determined gravimetrically using a preweighed eppendorf tube for bile collection. Results obtained in *μ*L/min/Kg b.wt, assuming specific gravity of bile, are 1.0 g/mL.

### 2.5. Histopathological Examination and Caspase 3 Immunohistochemical Staining

Slices of fixed liver tissues were routinely processed in ascending grades of alcohol, cleared in xylene, and embedded in paraffin wax; serial sections were made for Hematoxylin and Eosin (H&E) staining and immunohistochemical staining of caspase 3 using Thermo Fisher Scientific Caspase 3 Rabbit Polyclonal Antibody (Fremont, CA, USA).

### 2.6. Statistical Analysis

Data were statistically analyzed by one-way ANOVA to compare between different groups with control and EE & CPZ groups followed by unpaired *t*-test. For analysis of the effect of different fibrates with and without GW6471, two-way ANOVA was used making fibrate type the first factor and presence or absence of GW6471 as the second factor. Regression analysis and correlation coefficient were done for standard curves. Statistical analysis was generated using Minitab computer software version 16. All results were expressed as the mean ± SD. The level of significance was set at *P* ≤ 0.05.

## 3. Results

As shown in Table 1, treatment of rats with 17*α* ethinylestradiol (5 mg/Kg/d S.C.) and chlorpromazine (30 mg/Kg/d) orally for 5 days resulted in significant decrease in bile flow rate and significant increase in all serum biochemical parameters as well as hepatic MDA level as compared to control group (*P* < 0.05). Treatment of rats with fenofibrate 2 days before ethinylestradiol + chlorpromazine administration resulted in significant reduction in serum levels of ALP by 53.23%, GGT by 58.11%, AST by 16.48%, ALT by 16.09%, TBA by 71.4%, direct bilirubin by 52.5%, total bilirubin by 62.77%, IL-1*β* by 59.85%, and TNF*α* by 39.5% and by 69.99% in hepatic MDA level as well as significant increase in bile flow rate by 274.03% compared to EE & CPZ group (*P* < 0.05). While in bezafibrate group there was a significant reduction in ALP by 47.9%, GGT by 57.75%, AST by 34.95%, ALT by 24.798%, TBA by 62.75%, direct bilirubin by 47.5%, total bilirubin by 58.36%, IL-1*β* by 51.91%, TNF*α* by 43.02%, and hepatic MDA by 67.96% and a significant increase in bile flow rate by 254.26% compared to EE & CPZ group (*P* < 0.05). Gemfibrozil group resulted in significant reduction in all biochemical parameters by 54.79% in ALP, 71.22% in GGT, 36.69% in AST, 29.26% in ALT, 72.28% in TBA, 55% in direct bilirubin, 65.93% in total bilirubin, 60.96% in IL-1beta, and 37.7% in TNF*α* serum levels and significant decrease by 69.83% in hepatic MDA as well as significant increase in bile flow rate by 377.44% compared to EE & CPZ group (*P* < 0.05). Ursodeoxycholic acid (UDCA) (100 mg/Kg/d) also showed significant decrease in ALP, GGT, AST, ALT, TBA, direct, total bilirubin, IL-1*β*, TNF*α*, and hepatic MDA by 47.05%, 52.89%, 35.14%, 32.99%, 58.94%, 42.5%, 58.99%, 27.94%, 29.7%, and 23.63%, respectively, as well as significant increase in bile flow rate by 215.25% compared to EE & CPZ group (*P* < 0.05).

Pretreatment of EE & CPZ treated rats with PPAR*α* receptor antagonist (GW6471) 1 mg/kg/d i.p 30 min before any of the following drugs: fenofibrate, bezafibrate, or gemfibrozil resulted in a significant increase in all parameters except for bile flow rate showing significant decrease (*P* < 0.05) when compared with their corresponding non-GW6471 treated groups and control groups as presented in Table 2.

### 3.1. Histopathological Examination of Liver Tissue

**Figure 1:**
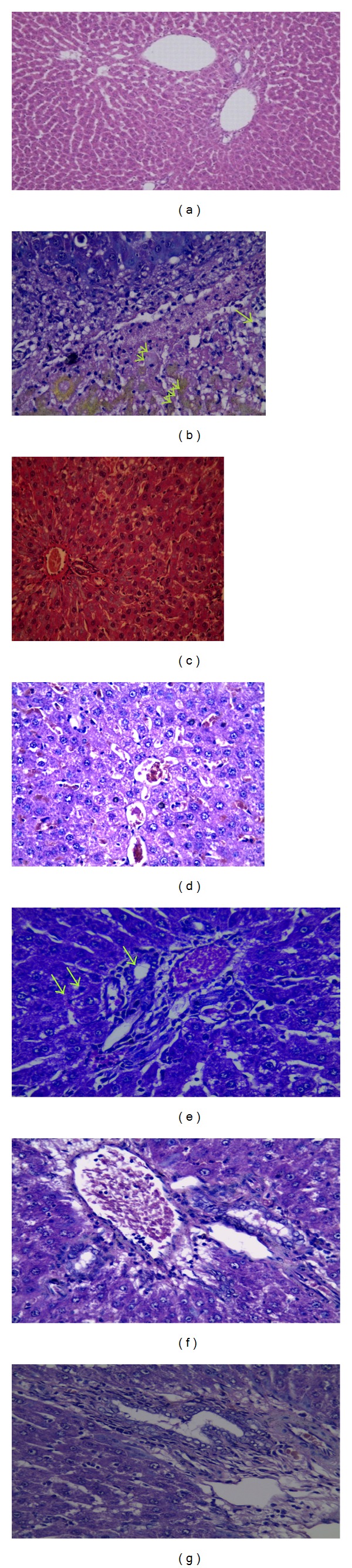
Histopathology of liver sections for groups 1–6: hematoxylin and eosin stain of liver tissue. (a) Control group showed normal hepatic architecture (H&E ×100). (b) Ethinylestradiol plus chlorpromazine treated group showed numerous apoptotic figures (pyknosis; one arrow and karyolysis; two arrows), intracellular bile pigments three arrows (H&E ×200). (c) Ethinylestradiol plus chlorpromazine treated group showed main bile duct obstruction, dilatation, and ductular proliferation (H&E ×100). (d) Ursodeoxycholic acid group showed ground glass cytoplasmic appearance of hepatocytes with congested dilated blood sinusoids (H&E ×200). (e) Fenofibrate group showed mild ground glass appearance of hepatocytes, proliferated bile ductules (one arrow) (H&E ×200). (f) Bezafibrate group showed proliferated bile ductules, congested central veins, and minimal mononuclear cellular infiltration (H&E ×200). (g) Gemfibrozil group showed mild hepatitic changes, portal tracts with mononuclear cellular infiltration, and proliferated bile ductules (H&E ×200).

Histopathological examination using H&E stained sections of liver samples of control group showed normal hepatic architecture, whereas examination of liver sections of animals treated with EE & CPZ showed numerous apoptotic figures, pyknosis and karyolysis associated with mononuclear cellular infiltration and green to yellowish brown areas of intracellular bile pigments (Figure 1(b)). Bile ducts were obstructed and dilated with ductular proliferation (Figure 1(c)).

Examination of liver sections of ursodeoxycholic acid (UDCA), fenofibrate, bezafibrate, gemfibrozil treated groups showed dilated and congested vascular bed; liver cells showed moderate to mild hepatitis (ground-glass cytoplasmic appearance) with scanty necrosis of some cells without intracellular brown pigments. These changes were only mild in gemfibrozil group.

**Figure 2:**
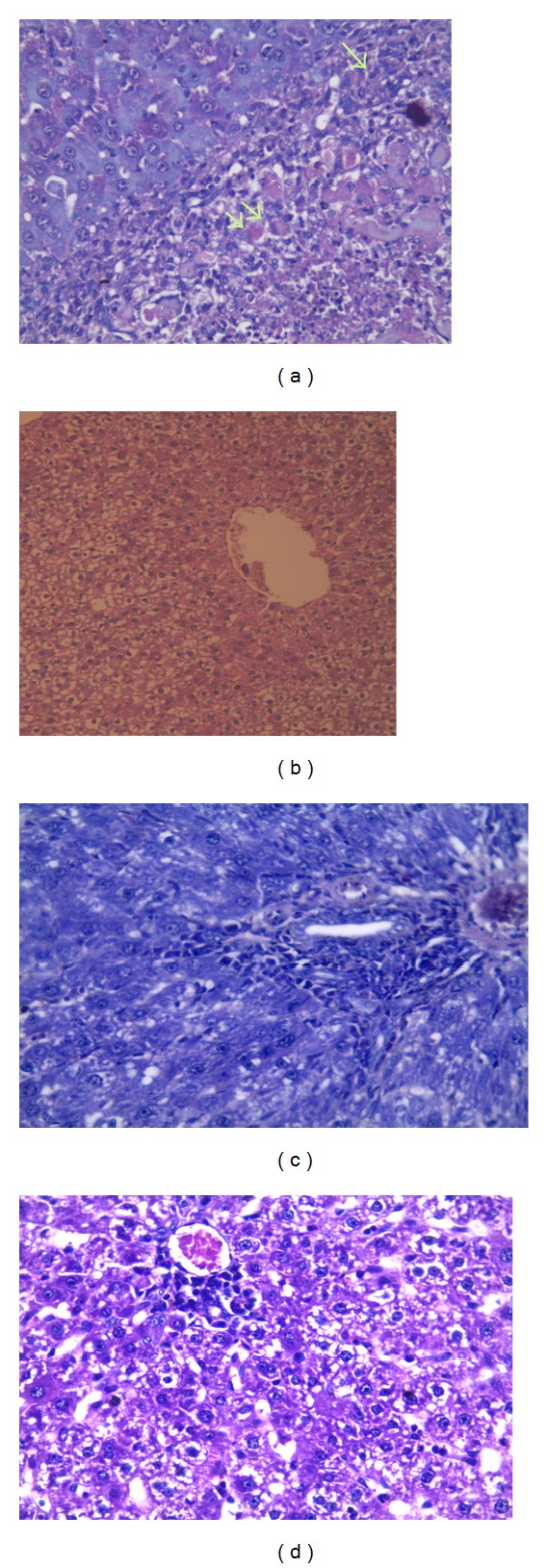
Histopathology of liver sections for groups 7–10: hematoxylin and eosin stain of liver tissue (H&E ×200). (a) Animals treated with GW6471 before fenofibrate and EE & CPZ showed highly eosinophilic cells (one arrow) and necrotic cells (two arrows) with dense mononuclear cellular infiltrations and hypertrophied kupffer cells. (b) Rats treated with GW6471 before fenofibrate and EE & CPZ showed dilated bile duct with no proliferation (H&E ×100). (c) Portal tract in rats treated with GW6471, bezafibrate, EE, and CPZ showed dense mononuclear cellular infiltration and moderate hepatitis changes (H&E ×100). (d) Animals treated with GW6471, gemfibrozil, EE, and CPZ showed dilated engorged central vein and blood sinusoid, mononuclear cellular infiltration, and vacuolar degeneration of liver cells.

On the other hand, examination of liver sections treated with EE & CPZ, fenofibrate, and GW6471 showed multiple necrotic highly eosinophilic cells, others with karyorrhexed nuclei, dense mononuclear cellular infiltration, and hypertrophied kupffer cells. No ductular proliferation was noticed (Figures 2(a) and 2(b)).

Whereas examination of liver sections of rats treated with GW6471 before bezafibrate and EE & CPZ showed mildly dilated central veins and blood sinusoids, hypertrophied kupffer cells, and moderate infiltration by mononuclear cells (Figure 2(c)), rats treated with EE & CPZ, gemfibrozil and GW6471 showed scattered focal necrotic cells, mononuclear cellular infiltration, and vacuolar degeneration with central venous and sinusoidal dilatation (Figure 2(d)).

### 3.2. Effects on Immunohistochemical Staining of Caspase-3 for Apoptosis Detection

**Figure 3:**
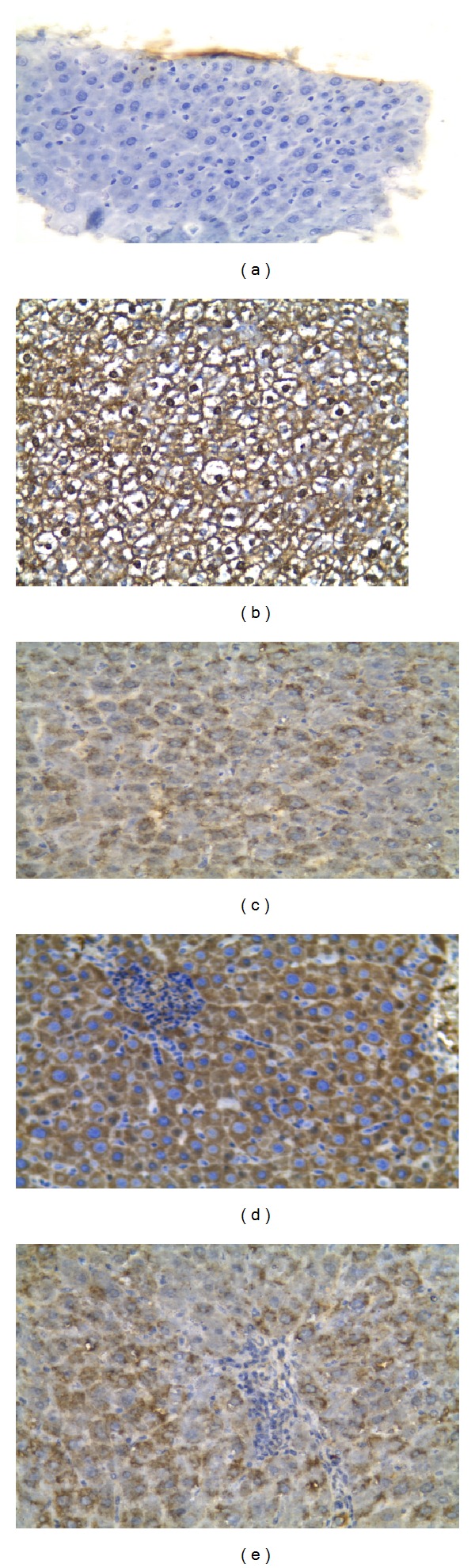
Caspase 3 immunohistochemical staining of apoptosis: immunohistochemical staining of Caspase 3 in liver tissues (PAP ×200) of (a) control animals showed negative (−) caspase 3 stain. (b) Highly positive (+++  +) for caspase 3 as in EE/CPZ group or fenofibrate plus GW6471 group. (c) Mild caspase 3 positivity (+) as in fenofibrate, bezafibrate, gemfibrozil, and UDCA groups. (d) Moderate cytoplasmic caspase 3 positivity (+++) as in bezafibrate plus GW6471. (e) Moderate positivity (++) in the cytoplasmic (granular) stain for caspase 3 as in gemfibrozil plus GW6471 group.

Immunohistochemical staining of sections for apoptosis detection was scored qualitatively as (−) for normal sections noticed in control group, (+) mild apoptosis as in fenofibrate, bezafibrate, gemfibrozil, and UDCA groups, moderate (++) as in gemfibrozil plus GW6471 group, (+++) as in bezafibrate plus GW6471, and severe apoptosis (+++  +) as in EE/CPZ group or fenofibrate plus GW6471 group (Figure 3).

## 4. Discussion

Cholestasis results from failure in bile secretion in hepatocytes or ductular cells or from a blockade to the free bile flow. 

Our article is the first one studying the effect of three different fibrates on EE & CPZ induced intrahepatic cholestasis and focusing on the mechanism of their effect and whether it is PPAR*α* dependent or not.

Although this is an experimental animal study, it may be of value in clinical management of pregnancy-induced intrahepatic cholestasis and cholestasis induced by contraceptive pills containing estrogen in addition to cholestatic patients receiving antipsychotic chlorpromazine.

In the present study simultaneous administration of EE plus CPZ resulted in a significant elevation in liver function tests as compared to normal control group as well as a significant decrease in bile flow rate compared to control animals in addition to a significant increase in hepatic MDA, serum TNF*α*, and IL-1*β* serum levels (*P* ≤ 0.05) revealing a convenient pathophysiological impact of EE-CPZ combination on hepatobiliary function. These biochemical results were in agreement with Said and El-Agamy, who used similar model of intrahepatic cholestasis [16].

Several studies revealed that EE increases cholesterol ester content of liver homogenate and decreases fluidity of cell membranes [17, 18]. This consequently decreases bile flow and Na^+^/K^+^-ATPase activity [5]. Moreover EE has been shown to decrease the uptake of bile acids and other organic anions into isolated hepatocytes [19]. Chlorpromazine has been reported to induce cholestatic hepatitis. The mechanism of CPZ-induced cholestasis is explained by its detergent properties which enable CPZ to bind to membrane phospholipids leading to alteration in membrane fluidity and inhibition of Na^+^/K^+^-ATPase activity [20]. Also, CPZ affects the polymerization of actin in actin-containing microfilaments which are responsible for the canalicular contraction and mobility thus leading to inhibition of normal canalicular bile secretion [21]. The cholestatic effect of EE was further enhanced by combination with CPZ.

Elevation of MDA contents in liver tissue indicated the implication of oxidative stress in hepatic tissue damage induced by EE-CPZ treatment. This result was consistent with other studies that showed the contribution of oxidative stress in the pathogenesis of cholestasis as a consequence of generation of CPZ cation radicals and/or metabolic activation of CPZ to quinoneimine derivatives [22] and decrease in hepatic super oxide dismutase SOD, glutathione peroxidase GPx, and glutathione reductase GR activity after EE administration [23]. 

In addition, histopathological examination of excised livers showed marked bile duct proliferation, marked inflammation, noticeable apoptotic figures, and yellowish brown bile pigment indicating cholestasis. These findings were in agreement with previous reports [16, 24, 25]. Also enhanced apoptosis is observed following EE/CPZ 5 days administration as shown by Caspase 3 staining. 

In the present study fenofibrate, bezafibrate, and gemfibrozil showed a significant decrease in biochemical parameters as well as a significant increase in bile flow rate relative to EE-CPZ treated animals (*P* < 0.05). These biochemical results were in agreement with previous studies using fenofibrate on extrahepatic cholestatic model [26, 27].

Although this study seems similar to that of Cindoruk et al. [26], this study proved fibrates' effect on intrahepatic cholestasis not on extrahepatic cholestasis as in the latter study. In addition, it studied the effects of three different commercially available fibrates not only fenofibrate.

Leuenberger et al. explained the ability of fenofibrate to decrease bilirubin serum level in EE treated animals by the ability of PPAR alpha ligands to repress CYP7b1 gene expression in male and female mice which was enhanced by estrogen [28].

This choleretic action of fibrates mainly bezafibrate was further attributed to enhancement in canalicular membrane fluidity (opposing EE and CPZ cholestatic mechanism discussed above) and transporter activity mediating bile acid-independent bile secretion [29]. 

The increased plasma transaminase levels of fenofibrate could be attributed to an increase in hepatic transaminase activities associated with an increase in hepatic transaminase genes and were not considered to be a consequence of hepatotoxicity from the drug [30]. 

The antioxidant effects of fibrates through decrease in MDA levels could be explained by several mechanisms. First, oxidative injury has decreased due to the increased level of antioxidant enzymes as a result of PPAR activation [31]. Second, several fibrates metabolites (but not fibrates themselves) possess direct radical scavenging properties [32]. Third, some studies demonstrated that treatment with fibrates reduces the susceptibility of plasma lipoproteins, especially LDLs, to oxidation [32, 33]. Fourth, fibrates have potent anti-inflammatory properties decreasing ROS generation by phagocytes [34]. This was noticed in the current study in the form of a decrease in serums IL-1*β* and TNF*α* as well as reduced portal inflammation and necrosis in histopathology. Finally, PPAR*α* agonists stimulate the expression of cytochrome p450, which catabolizes some lipid peroxidation products including hydroxynonenal [35].

The present study showed that short-term administration of three fibrates decreased apoptosis in EE/CPZ experimental model of cholestasis. PPAR*α* agonists activate nuclear factor kappa B (NF-*κ*B) in the rat and mouse liver but not in the hamster. It has also been shown that NF-*κ*B has an antiapoptotic activity in several cell types, including hepatic cell lines [36]. 

Clinical trials using fibrates showed beneficial effects on biochemical parameters and in part also on histological findings in patients with PBC [37–42]. However, these studies were pilot studies including only a small number of patients and were not randomized controlled trials. So, further clinical studies are recommended to investigate fibrate efficacy on cholestasis due to the difference in PPAR*α* expression between animals and human. 

In the present work, some differences among the three agonists in reducing cholestatic parameters were noticed.

Gemfibrozil resulted in the lowest levels of all parameters and the highest bile flow rate. However, these differences were only significant from other fibrates and from UDCA in ALP, GGT, and IL-1*β* levels and bile flow rate indicating the superiority as anticholestatic agent over other drugs, while bezafibrate showed the least effectiveness among fibrates.

Concerning histopathology, fibrates decreased liver injury, necrosis, apoptosis, and intracellular bile pigments accumulation. Liver sections of fibrates showed higher bile ducts proliferation than EE & CPZ group. This may be a compensatory mechanism for enhancing bile flow after main duct obstruction.

In the present study UDCA was used as a positive control (drug officially used for treatment of cholestatic diseases in human) for comparing its effect to that of different PPAR alpha agonists whose anticholestatic effects are being investigated.

Generally fibrates showed better anticholestatic effects revealed by biochemical parameters levels and bile flow rate compared to the commonly used anticholestatic drug, UDCA, especially when compared to gemfibrozil, and this was further confirmed by histopathology.

Although nuclear receptors other than PPAR*α* are suggested to be potential targets in cholestasis like FXR and PXR [43], there is interplay between different nuclear receptors; for example, there is crosstalk between the PPAR*α* and FXR transcriptional pathways because PPAR*α* is an FXR target gene harboring an FXR response element in its gene promoter [4]. Being commercially available drugs makes studies on fibrates (known as being PPAR*α* agonists) for cholestasis management with higher priority than potent FXR ligands under early clinical trials.

Due to the biochemical differences in anticholestatic activities among the three fibrates the current study tried to investigate the mechanisms of their effects and whether they are only PPAR*α* dependent or not. To examine this, a selective and irreversible PPAR*α* antagonist, GW6471, was used. The dose and route of administration of GW6471 was determined according to previous study [44].

Interestingly, the three fibrates showed different trends in prevention of EE-CPZ cholestasis in the presence of this PPAR*α* blocker.

Fenofibrate anticholestatic effect was completely blocked with GW6471 treatment and all biochemical parameters; bile flow rate and histopathological findings were reversed with no significant difference from group of EE-CPZ treated animals indicating that the anticholestatic action of fenofibrate was solely PPAR*α* dependent.

This result was in agreement with previous study that compared fenofibrate effect on EE cholestasis in wild type and PPAR*α* null mice [28]. 

The histopathological examination confirmed these results and showed marked necrosis, congestion, apoptosis, and severe inflammation in group treated with GW6471 before fenofibrate resembling those changes in EE/CPZ group. Increased activation of kupffer cells after fenofibrate/GW6471 treatment indicates not only inflammation but also the oxidative injury appearing biochemically as increased MDA level.

However, bezafibrate anticholestatic effect was partially blocked with GW6471 treatment as described by significant increase in biochemical parameters as well as significant reduction in bile flow rate compared to animals treated with bezafibrate alone and these changes were still significantly different from EE & CPZ group.

Histopathological findings of bezafibrate/GW6471 group revealed moderate changes regarding bile stasis, obstruction, and inflammation although these changes were significantly higher than group treated with bezafibrate without the blocker. The bile ducts populations were nearly normal indicating that the duct proliferative effect was PPAR alpha dependent.

These findings revealed that bezafibrate anticholestatic effect was not completely dependent on PPAR*α* agonism and that other mechanisms were involved, may be by induction of other PPAR isoforms *β*/*γ* as well. Furthermore, although many changes induced by bezafibrate were clearly more dependent on PPAR*α*, induction of some PPAR*α* target genes by bezafibrate could be modulated in the absence of a functional PPAR*α* using null mouse [45].

Recently, Iwasaki et al. have reported the hepatoprotective effect of PPAR*β*/*δ* selective ligand in bile duct ligated animal model and its ability to significantly reduce serums ALT, TNF*α*, and IL-1*β* levels [46].

Some articles have demonstrated that PPAR*γ* also could regulate bile acid homeostasis adding another possible non-PPAR*α* mechanism to bezafibrate action [47]. 

Regarding gemfibrozil in equimolar dose relative to fenofibrate and bezafibrate doses, the anticholestatic effect was not completely reversed that is partially blocked by GW6471 administration.

Although the affinity of gemfibrozil with PPAR*α* is much lower than fenofibrate and bezafibrate [48], this low affinity allows this drug to perform many other biological activities independent of PPAR*α* [49]. 

Although, the most common application of gemfibrozil is to reduce the plasma lipids, the critical impact of gemfibrozil on numerous diseases including atherosclerosis [50], diabetes [51], arthritis [52], cancer [53], and CNS disorders [54] could not be ignored. A number of basic, preclinical, and clinical studies proposed that gemfibrozil might be used as an immunomodulatory, anti-inflammatory, antioxidant, and antimigratory drug independent on PPAR*α* [55].

Comparing the results of gemfibrozil with and without the blocker, we can notice that non-PPAR*α* agonism factors play a vital role in gemfibrozil anticholestatic effect probably more than PPAR*α* agonist effect.

These findings were also affirmed through histopathological examinations which revealed scanty necrosis, mild inflammation, and vascular degeneration. Although these changes were higher than gemfibrozil treated group, they were noticeably lower than changes detected from EE/CPZ or from the full PPAR alpha agonist, fenofibrate after treatment with GW6471.

It is evident from histopathology that fibrates increased bile duct proliferation as a compensatory mechanism to main duct obstruction while this proliferation was not present in groups receiving the blocker indicating that bile duct proliferation was mainly due to PPAR*α* agonism.

Caspase 3 immunohistochemical staining (a selective technique for apoptosis detection) revealed that group of EE plus CPZ and group treated with fenofibrate and GW6471 showed severe cytoplasmic apoptosis much higher than groups treated with fenofibrate, bezafibrate, gemfibrozil, or UDCA indicating the effect of these drugs in apoptosis suppression. Bezafibrate and gemfibrozil groups pretreated with GW6471 showed moderate apoptosis indicating that non-PPAR*α* mechanism participates with apoptosis suppression with PPAR*α* activation.

## 5. Conclusion

Fibrates might be effective in prevention of intrahepatic cholestasis produced by estrogens and CPZ, and this effect was mainly due to PPAR*α* agonist mechanism; however, other mechanisms might play part in bezafibrate and gemfibrozil actions. These findings may open the way on the use of these drugs in human susceptible to intrahepatic cholestasis by estrogens like pregnant, postmenopausal women or women receiving oral contraceptives and for CPZ patients as well.

## Figures and Tables

**Table 1:** Effect of ursodeoxycholic acid, fenofibrate, bezafibrate, and gemfibrozil on serums ALP, GGT, ALT, AST, TBA, direct bilirubin, total bilirubin, IL-1*β*, TNF*α*, and hepatic MDA levels in EE and CPZ treated rats.

Parameter	Control	EE + CPZ	UDCA	Fenofibrate	Bezafibrate	Gemfibrozil
ALP U/L	192.99 ± 22.9	556.59 ± 69.5*	294.73 ± 58.9^a^	260.3 ± 51.4^a^	289.9 ± 41.8^a^	251.62 ± 34.1^a^
GGT U/L	6.26 ± 2.8	19.9 ± 5.4*	9.38 ± 3.76^a^	8.3 ± 2.3^a^	9.01 ± 2.26^a^	5.7 ± 1.5^a^
AST U/mL	62.34 ± 8.3	150.69 ± 15.6*	97.7 ± 5.09^a^	125.8 ± 20.55^a^	98.02 ± 11.18^a^	95.4 ± 10.07^a^
ALT U/mL	42.16 ± 7.66	85.5 ± 11.83*	57.3 ± 5.5^a^	71.77 ± 13.4^a^	64.3 ± 11.5^a^	60.5 ± 12.3^a^
TBA *μ*mole/L	14.5 ± 7.08	81.75 ± 20.2*	33.56 ± 1.45^a^	23.38 ± 6.24^a^	30.45 ± 1.46^a^	22.66 ± 3.16^a^
Direct Bil. *μ*mole/L	0.436 ± 0.07	2.065 ± 0.36*	1.1826 ± 0.304^a^	0.975 ± 0.324^a^	1.086 ± 0.33^a^	0.932 ± 0.333^a^
T. Bil. *μ*mole/L	1.47 ± 0.39	5.42 ± 1.39*	2.496 ± 0.48^a^	2.0197 ± 0.476^a^	2.359 ± 0.436^a^	1.85 ± 0.873^a^
IL-1*β* pg/mL	0.104 ± 0.02	0.27 ± 0.059*	0.196 ± 0.044^a^	0.109 ± 0.012^a^	0.13 ± 0.026^a^	0.106 ± 0.019^a^
TNF*α* ng/mL	0.093 ± 0.109	0.1648 ± 0.29*	0.1158 ± 0.223^a^	0.0997 ± 0.105^a^	0.0939 ± 0.05^a^	0.1026 ± 0.06^a^
MDA *μ*mole/gm tissue	20.432 ± 0.1	75.96 ± 12.84*	58.01 ± 20.45^a^	22.79 ± 6.455^a^	24.34 ± 9.3^a^	22.92 ± 5.5^a^
Bile flow rate *μ*L/min/Kg b·wt	25.69 ± 5.4	3.8675 ± 2.3*	12.12 ± 1.7^a^	14.465 ± 1.8^a^	18.48 ± 1.59^a^	13.7175 ± 1.8^a^

ALP: alkaline phosphatase; GGT: gamma glutamyl transpeptidase; AST: aspartate aminotransferase; ALT: alanine aminotransferase; TBA: total bile acids; Direct Bil: direct bilirubin; T. Bil.: total bilirubin; IL-1*β*: interleukin-1beta; TNF*α*: tumor necrosis factor alpha; MDA: malondialdehyde; EE: ethinylestradiol, CPZ: chlorpromazine; UDCA: ursodeoxycholic acid. Data are presented as mean ± SD. *Significantly different from control group *P* < 0.05, ^a^significantly different from EE + CPZ group *P* < 0.05.

**Table 2:** Effect of pretreatment of EE and CPZ treated rats with PPAR*α* receptor antagonist (GW6471) 1 mg/kg/d i.p 30 min before fenofibrate, bezafibrate, or gemfibrozil on biochemical parameters and bile flow rate.

Parameter	GW6471	EE + CPZ	Feno + GW	Beza + GW	Gem + GW
ALP U/L	226.9 ± 22.9^a^	556.59 ± 69.5^∗b^	514.98 ± 57.4^∗b∧^	493.66 ± 35.4^∗ab∧^	411.82 ± 22,6^∗ab∧^
GGT U/L	8.69 ± 1.3^a^	19.9 ± 5.4^∗b^	16.56 ± 4.85^∗b∧^	9.01 ± 2.25^∗ab∧^	15.97 ± 5.23^∗b∧^
AST U/mL	67.93 ± 9.6^a^	150.69 ± 15.6^∗b^	148.18 ± 20.96^∗b∧^	98.03 ± 11.19^∗a∧bF^	111.79 ± 19.04^∗ab∧F^
ALT U/mL	49.137 ± 7.99^a^	85.5 ± 11.83^∗b^	86.132 ± 11.97^∗b∧^	77.21 ± 13.36^∗ab∧^	73.33 ± 7.398^∗ab∧F^
TBA *μ*mole/L	16.116 ± 4.104^a^	81.75 ± 20.2^∗b^	79.65 ± 15.92^∗b∧^	72.85 ± 19.35^∗a∧bF^	61.69 ± 14.9^∗ab∧F^
Direct Bil. *μ*mole/L	0.9025 ± 0.316^a^	2.065 ± 0.36^∗b^	1.97 ± 0.418^∗b∧^	1.387 ± 0.28^∗a∧bF^	1.286 ± 0.329^∗ab∧F^
T. BIL. *μ*mole/L	1.97 ± 0.722^a^	5.42 ± 1.39^∗b^	5.637 ± 1.83^∗b∧^	3.786 ± 1.318^∗a∧bF^	3.353 ± 0.79^∗ab∧F^
IL-1*β* pg/mL	0.109 ± 0.013^a^	0.27 ± 0.059^∗b^	0.2435 ± 0.099^∗b∧^	0.1694 ± 0.043^∗ab∧^	0.1541 ± 0.037^∗ab∧F^
TNF*α* ng/mL	0.101 ± 0.09^a^	0.1648 ± 0.29^∗b^	0.155 ± 0.2^∗b∧^	0.0939 ± 0.05^∗ab∧F^	0.1232 ± 0.01^∗a∧bF^
MDA *μ*mole/gm tissue	29.15 ± 11.53^a^	75.96 ± 12.84^∗b^	69.38 ± 21.3^∗b∧^	59.67 ± 19.11^∗ba∧^	46.12 ± 15.9^∗ab∧F^
Bile flow rate *μ*L/min/Kg b·wt	19.87 ± 1.09^a^	3.8675 ± 2.3^∗b^	5.13 ± 1.33^∗b∧^	7.98 ± 1.34^∗ab∧F^	10.055 ± 1.02^∗ab∧F^

ALP: alkaline phosphatase; GGT: gamma glutamyl transpeptidase; AST: aspartate aminotransferase; ALT: alanine aminotransferase; TBA: total bile acids; Direct Bil: direct bilirubin; T. Bil.: total bilirubin; IL-1*β*: interleukin-1beta; TNF*α*: tumor necrosis factor alpha; MDA: malondialdehyde; EE: ethinylestradiol; CPZ: chlorpromazine; Feno: fenofibrate; Beza: bezafibrate; Gem: gemfibrozil; GW: GW6471. Data are presented as mean ± SD. *Significantly different from control, ^a^significantly different from EE and CPZ groups, ^b^significantly different from GW6471 group, ^∧^significantly different from corresponding group not receiving GW6471, ^F^significantly different from (feno + GW6471) group at *P* < 0.05.

## Abbreviations

PPAR*α*: Peroxisome proliferator-activated receptor alpha
CPZ: Chlorpromazine
GGT: Gamma glutamyl transpeptidase
AST: Aspartate aminotransferase
TNF*α*: Tumor necrosis factor alpha
MDA: Malondialdehyde
PPREs: Peroxisome proliferators response elements
S.C.: Subcutaneous
i.p: Intraperitoneal
SOD: Superoxide dismutase
GR: Glutathione reductase
NF-*κ*B: Nuclear factor kappa B
EE: Ethinylestradiol
ALP: Alkaline phosphatase
ALT: Alanine amino transferase
TBA: Total bile acids
IL-1*β*: Interleukin-1 beta
NRs: Nuclear receptors
Mdr-2: Multidrug resistance transporter-2
UDCA: Ursodeoxycholic acid
H&E: Hematoxylin and Eosin
GPx: Glutathione peroxidase
LDL: Low density lipoprotein.
